# 3D cell culture using a clinostat reproduces microgravity-induced skin changes

**DOI:** 10.1038/s41526-021-00148-6

**Published:** 2021-06-01

**Authors:** Dong Hyun Choi, Byoungjun Jeon, Min Hyuk Lim, Dong Hun Lee, Sang-Kyu Ye, Seung-Yong Jeong, Sungwan Kim

**Affiliations:** 1 Department of Biomedical Engineering, Seoul National University College of Medicine, Seoul, Korea; 2 Department of Emergency Medicine, Seoul National University College of Medicine, Seoul National University Hospital, Seoul, Korea; 3 Interdisciplinary Program in Bioengineering, Graduate School, Seoul National University, Seoul, Korea; 4 Department of Dermatology, Seoul National University College of Medicine, Seoul, Korea; 5 Institute of Human-Environment Interface Biology, Seoul National University, Seoul, Korea; 6 Department of Pharmacology and Biomedical Sciences, Seoul National University College of Medicine, Seoul, Korea; 7 Department of Surgery, Seoul National University College of Medicine, Seoul, Korea; 8 Institute of Bioengineering, Seoul National University, Seoul, Korea

**Keywords:** Cell biology, Biophysics

## Abstract

Exposure to microgravity affects human physiology in various ways, and astronauts frequently report skin-related problems. Skin rash and irritation are frequent complaints during space missions, and skin thinning has also been reported after returning to Earth. However, spaceflight missions for studying the physiological changes in microgravity are impractical. Thus, we used a previously developed 3D clinostat to simulate a microgravity environment and investigate whether physiological changes of the skin can be reproduced in a 3D in vitro setting. Our results showed that under time-averaged simulated microgravity (taSMG), the thickness of the endothelial cell arrangement increased by up to 59.75%, indicating skin irritation due to vasodilation, and that the diameter of keratinocytes and fibroblast co-cultured spheroids decreased by 6.66%, representing skin thinning. The α1 chain of type I collagen was upregulated, while the connective tissue growth factor was downregulated under taSMG. Cytokeratin-10 expression was significantly increased in the taSMG environment. The clinostat-based 3D culture system can reproduce physiological changes in the skin similar to those under microgravity, providing insight for understanding the effects of microgravity on human health before space exploration.

## Introduction

During spaceflight, astronauts are exposed to microgravity, which has various effects on human physiology. Bone and muscle mass are decreased, brain structures are altered, and intravascular fluid is redistributed after exposure to microgravity^1–3^. One of the organs that is most affected is the skin. In a review of the electronic medical records of long-duration International Space Station (ISS) crew members, skin rashes and hypersensitivity were the most frequently reported conditions^4^. The National Aeronautics and Space Administration (NASA) found that skin irritation and rash were the most frequently reported complaints during ISS missions, with a 0.69 person-year incidence for both complaints^5^. Dermatitis, skin peeling, and infection have also been reported as dermatology disorders occurring in space^6,7^.

Skin thinning and loss of elasticity occur in astronauts after space flight^8^. Similar results were observed in a study of mice which spent 3 months in the ISS. The dermal thickness of mice exposed to prolonged microgravity was reduced by 15% compared to that of controls^9^. In the same study, the authors found that collagen turnover was increased.

Endothelial cells, which are the major component of dermal blood vessels, are also affected by microgravity. Shi et al. reported that one day of exposure to simulated microgravity (SMG) promotes angiogenesis^10^. Carlsson et al. found that microgravity stimulated endothelial cell growth and changed the morphology of the cellular structure^11^. Altered angiogenesis has been implicated in chronic inflammatory skin disorders such as atopic dermatitis and psoriasis as well as skin irritation, which is the most frequently reported skin problem in astronauts^5,12–14^.

Conducting a spaceflight mission to study the physiologic changes in microgravity is impractical because of the high cost and inaccessibility. To overcome these limitations, clinostats have been developed to simulate microgravity environments^15^. A 3D clinostat is constructed from two perpendicular frames that rotate independently. The direction of the gravity vector is constantly changed so that the average of the gravity vector simulates a microgravity environment. We previously developed a 3D clinostat and validated its performance^16^. Using a novel algorithm, the clinostat generated an evenly distributed time-averaged SMG (taSMG) condition.

Many studies have focused on the effects of space travel using 2D culture^10,17,18^. However, in vitro mimicry studies that directly compared cellular changes using 3D culture technique in 1 G and SMG environments were insufficient and limited because of differences in the culture settings of SMG. Some characteristics of cells cannot be modeled in 2D culture, and 3D culture such as spheroids or organoids can better reproduce responses that occur in vivo^19,20^. Scaffold-based 3D skin equivalent models, which represent the actual skin in a more structurally closer and physiological way, have also been developed but can be more costly^21^. According to various studies using 3D models, the increased dimension of the extracellular matrix around the cell can significantly affect cell properties such as interactions between cells, morphology, proliferation, mechanical response, etc^22,23^. Thus, this study was conducted to use a previously developed 3D clinostat to simulate a microgravity environment and investigate whether physiological changes of the skin can be reproduced in a 3D in vitro setting.

## Results

### Skin model

Human epidermal keratinocytes HaCaT, human skin fibroblasts Hs27, and human umbilical vein endothelial cells (HUVECs) were used as substitutes for epithelial cells, fibroblasts, and blood vessel endothelial cells present in the skin tissue. Cells were cultured in a 3D clinostat to observe the changes when exposed to taSMG.

### Effects of taSMG on 3D arrangement of endothelial cells

**Fig. 1:**
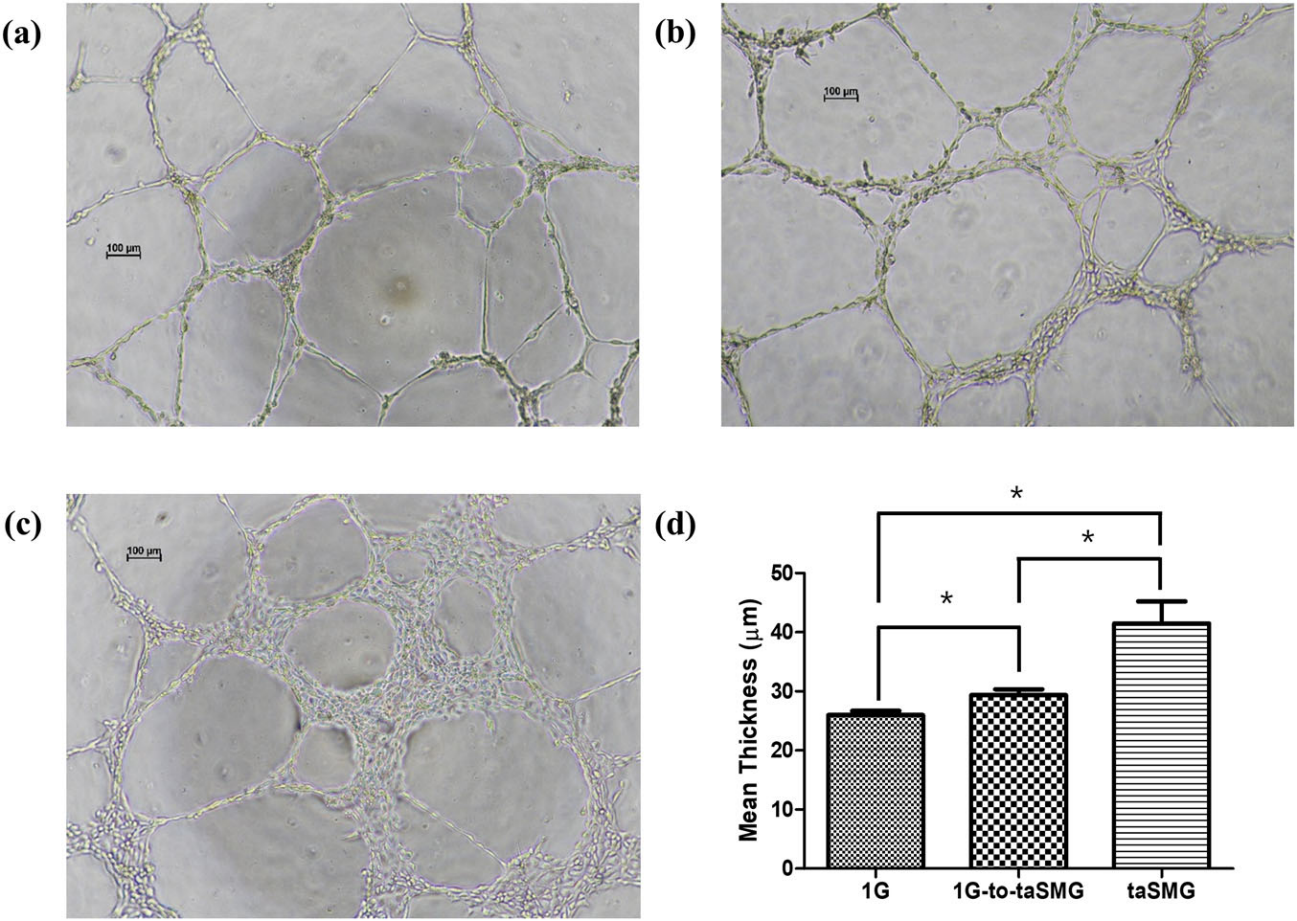
Arrangement of HUVECs under various gravity environments using Matrigel. After 18 h of incubation under **a** 1 G, **b** 1G-to-taSMG, and **c** taSMG. Scale bars: 100 μm. **d** Quantitative comparison of thickness between groups. Data represent the mean ± SEM (*n* = 8–9, *p < 0.05).

The arrangement of endothelial cells was thicker in Matrigel under a taSMG than under the 1 G environment (Fig. 1). The 1G-to-taSMG group, which was incubated at 1 G and then transferred to taSMG, also showed an increase in thickness compared to the 1 G group (Fig. 1d). The thicknesses under 1G-to-taSMG and taSMG were increased by 12.92% (*p* = 0.04) and 59.75% (*p* = 0.01) over 1 G, respectively. The thicknesses of arranged endothelial cells in each group were significantly different compared with each other (*p* = 0.03). These findings suggest that taSMG improves the ability of HUVECs to reconstruct into thicker arrays.

### Diameter of co-cultured spheroids decreased under taSMG

**Fig. 2:**
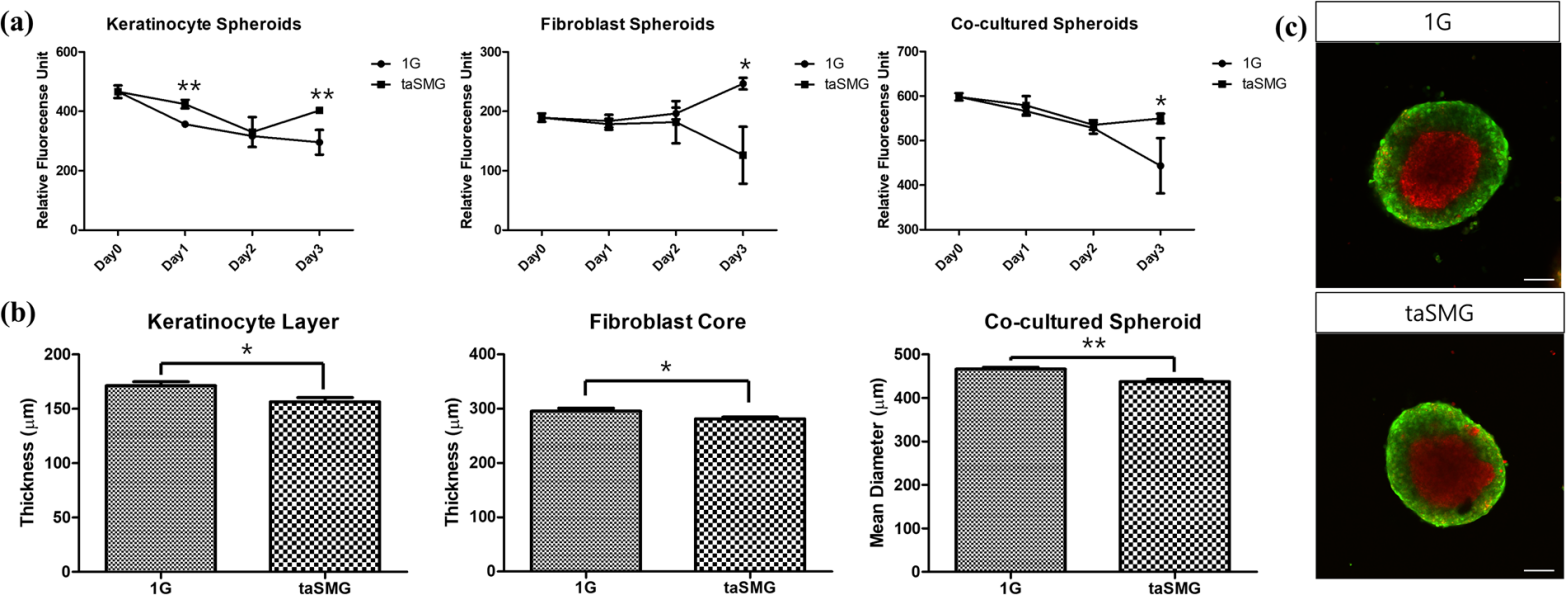
Cell viability and thickness of spheroids under 1 G and taSMG. **a** Cell viability of mono- and co-cultured spheroids for 3 days of culture. Keratinocyte spheroids showed significant difference at day 1 and 3. All spheroids showed a significant difference after 3 days. **b** Thickness between keratinocyte layer and fibroblast core and overall diameter of co-cultured spheroids. All comparisons between 1 G and taSMG showed significant differences after 3 days. Data represent the mean ± SEM (*n* = 8, *p < 0.05, **p < 0.01). **c** Confocal images of CellTracker^TM^ stained spheroids under 1 G and taSMG. Red tracker indicates fibroblast core and green tracker indicates keratinocyte layer. Scale bars: 100 μm.

Increased viability of keratinocytes and co-cultured spheroids was observed under taSMG, whereas the viability of fibroblast spheroids under taSMG was decreased (Fig. 2a). Similar results were observed in the typical 2D culture setup, except that the viability of fibroblasts was increased under taSMG (Supplementary Fig. 1). The thickness of the keratinocyte layer and fibroblast core decreased in the taSMG environment, resulting in a smaller diameter of co-cultured spheroids (Fig. 2b). The size of keratinocytes in co-cultured spheroids was shown to be significantly reduced in taSMG environment as shown in Supplementary Fig. 2 (*p* < 0.05). The thicknesses of the keratinocyte layer and fibroblast core were reduced by 5.11% (*p* = 0.02) and 9.44% (*p* = 0.05), respectively. The total diameter of the co-cultured spheroids under taSMG decreased by 6.66% (*p* < 0.01). CellTracker^TM^-stained keratinocytes (green) and fibroblasts (red) were distinguishable, as shown in Fig. 2c.

### Effects of taSMG on extracellular matrix homeostasis of co-cultured spheroids

**Fig. 3:**
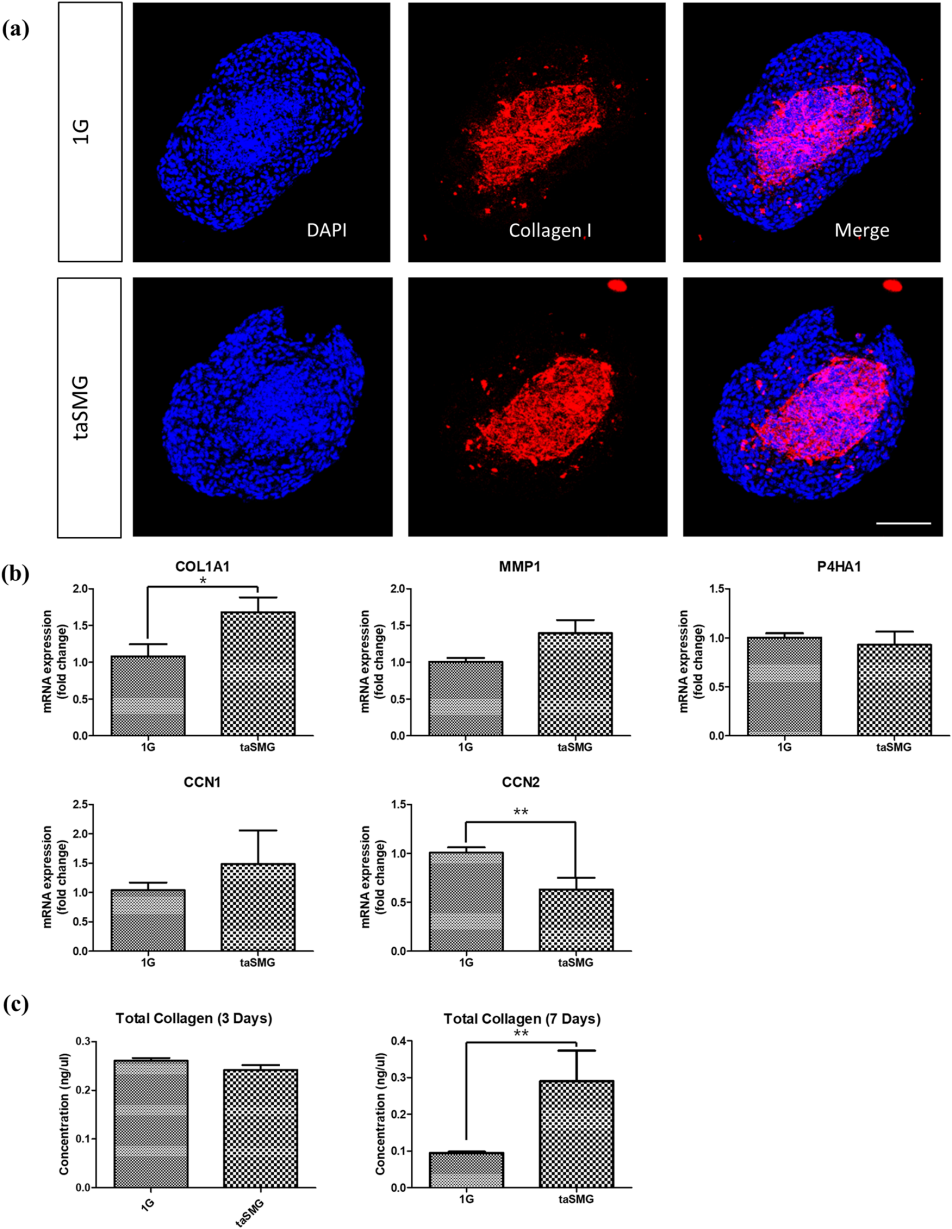
Extracellular matrix homeostasis of co-cultured spheroids under 1 G and taSMG. **a** Collagen type I and DAPI staining showing collagen expression in the fibroblast core and both nuclei of the keratinocyte layer and fibroblast core. Scale bar: 100 μm. **b** qRT-PCR analysis of COL1A1, MMP-1, P4ha1, CCN1, and CCN2. **c** Hydroxyproline assay result indicating collagen contents in spheroids after 3 and 7 days. Data represent the mean ± SEM (*n* = 4, *p < 0.05, **p < 0.01).

Collagen type I was stained in the fibroblast core of co-cultured spheroids as shown in Fig. 3a. The α1 chain of type I collagen (COL1A1) was upregulated in the taSMG group and showed a significant difference with the 1 G group. On the other hand, the connective tissue growth factor (Ctgf/CCN2) was significantly downregulated in the taSMG group (Fig. 3b). The expression of other genes showed no significant difference between the two groups. Interestingly, the expression of the same set of genes in 2D culture showed a significant increase under taSMG (Supplementary Fig. 3). The taSMG environment did not significantly affect the hydroxyproline content after 3 days but increased significantly after 7 days (Fig. 3c).

### Effects of taSMG on epidermal cytokeratin-10 of co-cultured spheroids

**Fig. 4:**
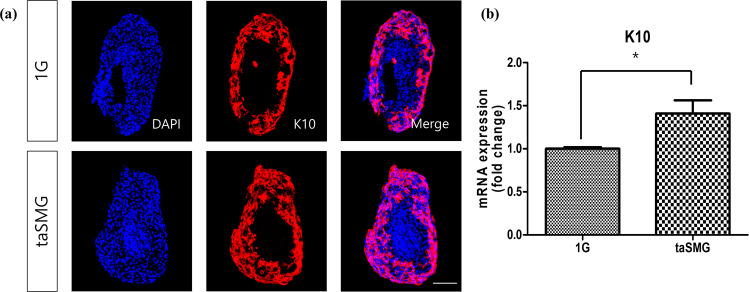
Cytokeratin-10 (K10) expression of co-cultured spheroids under 1 G and taSMG. **a** K10 and DAPI staining showing the cytokeratin expression in keratinocyte layer and both nuclei of the keratinocyte layer and fibroblast core. Scale bar: 100 μm. **b** qRT-PCR analysis of K10. Data represent the mean ± SEM (*n* = 4, *p < 0.05).

Cytokeratin-10 (K10) was stained in the keratinocyte layer of co-cultured spheroids (Fig. 4a). K10 expression was significantly increased in the taSMG environment (*p* = 0.04) (Fig. 4b). A significant increase in K10 expression was also observed in the 2D culture environment (Supplementary Fig. 3).

## Discussion

Alterations in physiological properties in space have been broadly reported, with the literature suggesting that biological changes occur within the cellular environment of human cells exposed to microgravity^4,24^. Astronauts repeatedly reported that they suffer from skin-related problems according to a study by the NASA^5^. Thinning of the skin layer and alteration in collagen contents have been reported in a space mouse model^9^. These findings strongly suggest that the physiology and structure of the skin are altered by microgravity. However, the effects of microgravity at the cellular level are not well-understood. Thus, we reproduced physiological changes in skin cell-based in vitro spheroids under taSMG using a previously developed clinostat to improve the understanding of gravitational effects on 3D cultured cells.

To investigate whether taSMG can induce in vitro angiogenesis, which has been implicated in chronic skin inflammatory disorders and skin irritation, a tube formation assay was conducted. As shown in Fig. 1d, the thickness of endothelial cell arrangement formed under the taSMG environment was significantly increased. The observed increase in thickness under taSMG can explain the potential for skin irritation because the dilation and increased permeability of blood vessels produces erythema and edema, which are hallmarks of skin irritation^25^. Thus, microgravity-exposed skin may be vulnerable to skin irritation and inflammatory skin disorders via increased angiogenesis. Additionally, the facial fullness and headache reported by astronauts may be related to the increased permeability or dilation of blood vessels during space adaptation, as taSMG increased the thickness of endothelial cell arrangement^5,8,26^.

Skin thinning is another phenomenon observed under microgravity^8,9^. The previously investigated mouse model showed a significant reduction in the dermal thickness similar to our results showing that the dermal component area of the spheroid was significantly reduced in size (Fig. 2)^9^. Further, skin physiology research on astronauts indicated thinning of the dermal matrix, which may be related to the expression of MMP-1 and CCN2^8^. Reduced expression of CCN2 has been shown to cause defective adhesion to fibronectin, which plays a major role in cell adhesion and maintenance of the cellular structure and shape, among other functions^27,28^. The previously reported thinning of the astronaut’s dermal matrix may be associated with fibronectin impairment, as indicated by the results showing that taSMG reduced CCN2 expression (Fig. 3b). Interestingly, the expression of COL1A1, which forms part of a large molecule known as type I collagen, was significantly upregulated (Fig. 3a, b). The total collagen contents determined in the hydroxyproline assay at day 7 were significantly increased which can be explained by the upregulation of COL1A1 (Fig. 3c). Collagen expression by fibroblasts requires a sufficient period of time after seeding and is highly dependent on the 3D-surrounding. Therefore, the absence of a significant change in collagen content at day 3 seems to be due to the short experiment time. Our findings show that the SMG environment generated by the clinostat can reproduce dermal thinning and induce changes in gene expression compared to those in a normal gravitational environment. In a previous study, CCN1 and CCN2 expression was shown to be upregulated in an actual microgravity environment, which contrasts our findings^9^. In another study using thyroid carcinoma spheroids, the expression of CCN2 was downregulated, similar to that in our study. The first study was limited by the small number of mice, resulting in questionable statistical reliability. However, we cannot exclude the possibility of a difference in gene expression between the taSMG and real microgravity environments. Therefore, further studies are required to fully understand this difference.

The decrease in thickness of the keratinocyte layer of spheroids under taSMG is similar to the decrease in the thickness of the stratum corneum of an astronaut (Fig. 2b). However, in co-cultured spheroids, the thickness of the keratinocyte layer decreased and the viability of keratinocytes increased (Fig. 2). This unexpected result could be related to the size of keratinocytes in co-cultured spheroids. As can be seen in Supplementary Fig. 2, the reduction in the size of keratinocytes in taSMG environment would have resulted in the formation of more dense structures and increasing viability while decreasing the thickness. Moreover, K10 expression was significantly increased under taSMG (Fig. 4b). K10 is important for maintaining epidermal homeostasis, and its expression is known to increase following acute and chronic skin barrier disruption^29,30^. Our findings suggest that the taSMG environment induced skin barrier disruption; however, further studies are needed to fully explain this phenomenon. Overall, our results indicate that taSMG produced by the 3D clinostat can reproduce the reported physiological and structural changes of skin components and enable investigation under microgravity conditions.

This study had several limitations. First, our co-cultured spheroid model only consists of two cell types and therefore is not completely identical to real skin. Also, the 3D culture of fibroblast cells is a simulation of one of the dermal cells of the skin, but cannot represent the dermal layer itself. Although co-cultured spheroids under taSMG can partially reproduce reported effects and physiological modulation, the culture environment must be further optimized. This will enable analysis of various cell types under taSMG. Organ-on-a-chip-based culture platform may be applied to the 3D clinostat environment to further investigate various phenomena within the chip models under taSMG. A microfluidic chip model would provide a better nutritional supply for the cells, spheroids, or organoids for a longer experimental period. Second, we examined spheroids and endothelial cell arrangement for only 3 days and 24 h, respectively. However, long-term ISS stays can span several months. There may be some limitations when explaining cellular changes beyond the time period of our experiment. However, for practical reasons, most previous studies observing changes in cells cultured under SMG were performed for less than a week^10,18^. Third, HaCaT is an immortalized keratinocyte cell line and does not reflect some features of normal human keratinocytes. HaCaT cells were used in this study to overcome the difficulties of culturing primary keratinocytes due to their short lifetime, donor-to-donor variability, and changes in characteristics according to the number of passages^31,32^. Although they were widely used as a reliable substitute for human keratinocytes in previous studies, this limitation should be considered when interpreting the results of this study.

Although improvements in the culture protocol are needed, our taSMG in vitro culture system reproduced physiological modulation of the skin components similar to that observed in astronauts and mouse models. Experiments using the proposed system may improve the understanding of the effects of microgravity on human health prior to space exploration.

## Methods

### Clinostat setup

The 3D clinostat was developed and validated in our previous research^16,33^. The 3D clinostat provides taSMG less than 10^-3^G. 1 G is the acceleration on earth due to gravity and is equal to 9.806 m/s^2^. Coriolis, radial, and frictional forces were not considered due to sufficiently small angular velocities of two axes (inner frame: 0.683 rpm, outer frame: 0.913 rpm). Operating the 3D clinostat at the designed angular velocity avoids repetitive patterns of gravitational vector, as demonstrated in previous research^16,33^.

### Cell culture

Human epidermal keratinocytes HaCaT (Addexbio, San Diego, CA, USA), a spontaneously transformed immortal human keratinocyte cell line, and human skin fibroblasts Hs27 (CRL1735, ATCC^®^, Manassas, VA, USA) were cultured in high-glucose DMEM (WELGENE, Daegu, Korea) supplemented with 10% fetal bovine serum (Gibco, Grand Island, NY, USA) and 1% penicillin/streptomycin solution (Gibco). The HUVECs (PCS-100-01, ATCC^®^) were cultured in EGM-2 supplemented with ascorbic acid, vascular endothelial growth factor, 20% FBS, recombinant human Fb growth factor, hydrocortisone, insulin-like growth factor-1, a recombinant analog of human insulin-like growth factor-1, gentamicin–amphotericin, and heparin (Lonza, Basel, Switzerland). HaCaT, Hs27, and HUVECs were used as substitutes for epithelial cells, fibroblasts, and blood vessel endothelial cells present in the skin tissue. All experiments used cells from passages 3–10. All cells were incubated in 5% CO_2_ at 37 °C.

### In vitro 3D arrangement of endothelial cells

Matrigel matrix (Corning, Inc., Corning, NY, USA) was incubated overnight on ice at 4 °C prior to the assay. On the day of the assay, the Matrigel matrix was transferred into a 96-well plate and allowed to polymerize for 1 h at 37 °C. The HUVEC suspension (150 µL) at 2 × 10^5^ cells/mL was seeded and incubated for 2 h at 37 °C and 5% CO_2_ to allow the HUVECs to attach onto the Matrigel. An additional 200 µL of medium was added to each well and the plate was sealed with AeraSeal film (Sigma, St. Louis, MO, USA). Group 1 G and taSMG were grown for 18 h in a humidified 37 °C, 5% CO_2_ incubator. Group 1G-to-taSMG was incubated under 1 G for 9 h and then incubated under taSMG for another 9 h. Arrangement of the endothelial cell was observed with an Eclipse Ts2 (Nikon, Tokyo, Japan) microscope under 4x magnification.

### Spheroid cultures

Spheroids were prepared in 96-well ultra-low attachment surface plates (Corning). To prepare mono-culture spheroids, fibroblasts (10,000 cells/well) and keratinocytes (10,000 cells/well) were seeded into the plates. For skin co-cultures, 10,000 each of fibroblasts and keratinocytes were added per well, with keratinocytes added at 24 h after formation of the fibroblast core. The mono- and co-cultures were sealed with AeraSeal film and cultured for 3 days under 1 G and taSMG. Spheroid formation was observed with an Eclipse Ts2 microscope under 4x magnification.

### Cell viability

The cell viability assay was performed using PrestoBlue HS cell viability reagent (Thermo Fisher Scientific, Waltham, MA, USA). PrestoBlue HS reagent (20 μL) was added to each well containing 200 μL of medium using a multichannel pipette and gently mixed by pipetting 2–3 times. Wells containing only fresh medium and the reagent were used as normalization controls (blank). The plate was incubated at 37 °C for 4 h, after which fluorescence was measured with a multi-detection microplate reader (SpectraMax M5, Molecular Devices, Sunnyvale, CA, USA) at excitation and emission wavelengths of 560 and 590 nm, respectively.

### Spheroid thickness

To distinguish between the different cells and measure the difference in spheroid thickness, CellTracker^TM^ Fluorescent Probes (Life Technologies, Carlsbad, CA, USA) were used. Before adding cells to the 96-well plates, fibroblasts were labeled with Cell-Tracker^TM^ Red CMPTX dye (Life Technologies, C34552) and keratinocytes were labeled with Cell-Tracker^TM^ Green CMFDA (Life Technologies, C2925). Groups of spheroids were exposed to either 1 G or taSMG and imaged using a confocal microscope (TCS SP8, Leica, Wetzlar, Germany). When the size of cells was measured, Cell-Tracker^TM^ Red CMPTX dye was used to label cells. The diameter of mono- and co-cultured spheroids and the size of cells were measured using ImageJ 1.53c software (NIH, Bethesda, MD, USA).

### Immunofluorescence staining

Spheroids were collected into an Eppendorf tube, washed once with PBS, and fixed with 4% paraformaldehyde at room temperature for 30 min. The spheroids were then incubated overnight at 4 °C in 20% sucrose in PBS. On the next day, samples were embedded in Tissue-Tek^®^ OCT compound (Sakura, Torrance, CA, USA) and cut with a CM-1950 cryostat (Leica Biosystems) to prepare 10-μm-thick sections. All sections were permeabilized with 0.1% Triton X-100 (Sigma-Aldrich) in PBS, blocked with 5% BSA (Genedepot, Katy, TX, USA) in 0.03% Tris-buffered saline containing 0.03% Tween 20, and stained with rabbit anti-collagen I (Abcam, ab34710, Cambridge, UK) and mouse anti-cytokeratin 10 (K10) (Thermo Fisher Scientific, MA5–13705), followed by goat anti-mouse Alexa Fluor 647 (Abcam, ab150115) or goat anti-rabbit Alexa Fluor 647 (Abcam, ab150075) secondary antibody labeling. Nuclei were stained with DAPI (Sigma-Aldrich). Finally, the sections were washed with PBS and mounted with mounting medium for confocal microscopy (TCS SP8, Leica).

### Hydroxyproline assay for collagen quantification

Spheroids were co-cultured in the high glucose DMEM supplemented with ascorbic acid for collagen quantification purposes. Co-cultured spheroids were hydrolyzed with 12 N HCl for 3 h and filtered with activated charcoal by centrifugation at 10,000 × *g* for 3 min. Hydroxyproline was then determined by measuring the absorbance at 560 nm according to the manufacturer’s instructions.

### Quantitative real-time PCR

Total RNA of co-cultured spheroids was isolated using TransZol Up reagent (TransGen Biotech, Beijing, China) according to the manufacturer’s instructions. Total RNA concentrations were determined using a NanoDrop™ one UV–Vis Spectrophotometer (Thermo Fisher Scientific). For reverse transcript PCR, samples were reverse-transcribed with oligo (dT) 18 using a thermal cycler (C-1000 Touch, Bio-Rad, Hercules, CA, USA). Quantitative real-time PCR was performed using mixtures containing SYBR™ Green master mix (Thermo Fisher Scientific) and the appropriate primers (Table 1) with a CFX Connect instrument (Bio-Rad). The PCR cycling conditions were as follows: 50 °C for 2 min, 95 °C 3 min, 45 cycles of 95 °C for 15 s, 60 °C for 30 s, and 72 °C for 1 min. GAPDH was used as an internal reference. The 2^-∆∆Ct^ method was used for relative quantification of gene expression^34^.

**Table 1:** Primer list for quantitative real-time PCR.

Gene	Primer sequence (5′–3′)
GAPDH	F: ATGACATCAAGAAGGTGGTGAA
	R: GCTGTTGAAGTCAGAGGAGAC
COL1A1	F: CCTGTCTGCTTCCTGTAAACT
	R: GTTCAGTTTGGGTTGCTTGTC
P4HA1	F: CAGATGTGCCTAGGTGTTCT
	R: CACCACAGAGGAGCTAAAAG
MMP1	F: CTCTGACATTCACCAAGGTCTC
	R: GATTTCCTCCAGGTCCATCAAA
CCN1	F: CTGGTATCTCCACACGAGTT
	R: CCAGCGTAAGTAAACCTGAC
CCN2	F: GAAGATGTACGGAGACATGG
	R: CAGTGTCTGGGGTTGATAGA
K10	F: AGCTCAAGCAAGCACTACTC
	R: CTAATCCTCCATAGCCACCT
F, forward; R, reverse.	

### Statistical analysis

All numeric data were processed using Microsoft Excel 2013 (Redmond, WA, USA) and analyzed using SPSS Statistics version 25 software (SPSS, Inc., Chicago, IL, USA). Images were quantitatively analyzed using ImageJ 1.53c software. Values were plotted using GraphPad Prism 5 and graphs are presented as mean ± standard error of the mean (SEM). All data were statistically analyzed by Student’s *t* test or Mann–Whitney U test or ANOVA. Two sided *p*-values are indicated as *p < 0.05 and **p < 0.01.

### Reporting summary

Further information on research design is available in the Nature Research Reporting Summary linked to this article.

## Supplementary information

Supplementary Information

Reporting Summary

## Data Availability

All data generated or analyzed during this study are included in this published article or if not are available from the corresponding author on reasonable request.
