# Supplementary material for: 3D cell culture using a clinostat reproduces microgravity-induced skin changes
Source: NPJ Microgravity. 2021 Jun 1;7:20. doi: 10.1038/s41526-021-00148-6 (PMC8169764; doi:10.1038/s41526-021-00148-6)
Supplement: Supplementary file 1 — Supplementary Information [file 41526_2021_148_MOESM1_ESM.pdf]

Supplementary Figures

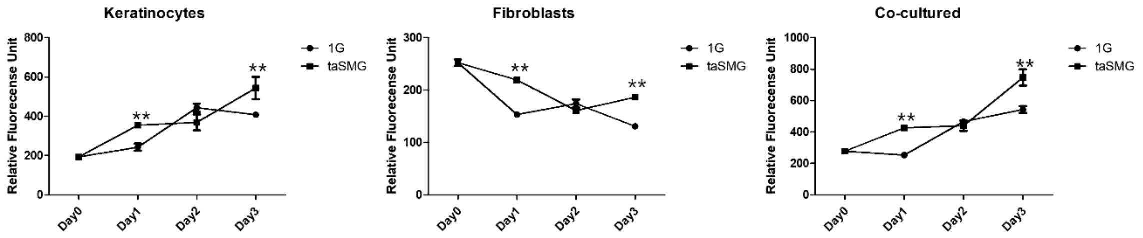

Supplementary Figure 1. Cell viability of keratinocytes, fibroblasts, and co-cultured cells under 1G and taSMG for 3 days of culture. All cells showed a significant difference on days 1 and 3. Data represent the mean ± SEM (n = 8, *p < 0.05, **p < 0.01).

(a) (b)

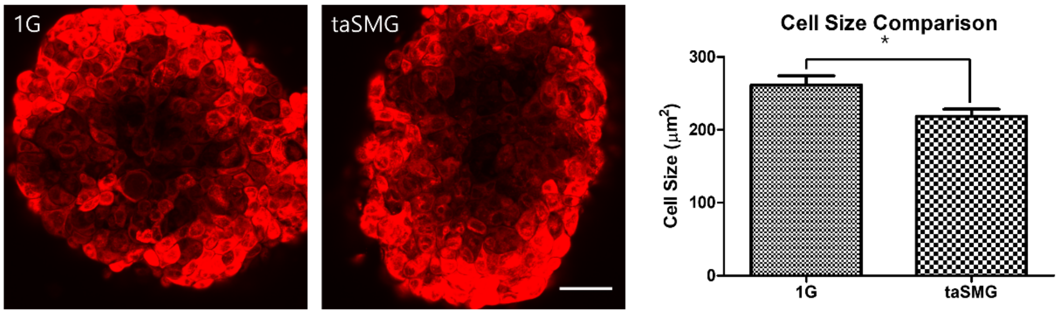

Supplementary Figure 2. Size of keratinocytes in spheroids. (a) CellTracker™-stained spheroids. Scale bars: 50μm. (b) Quantitative comparison of cell sizes under 1G and taSMG conditions. Data represent the mean ± SEM (n = 3, *p < 0.05).

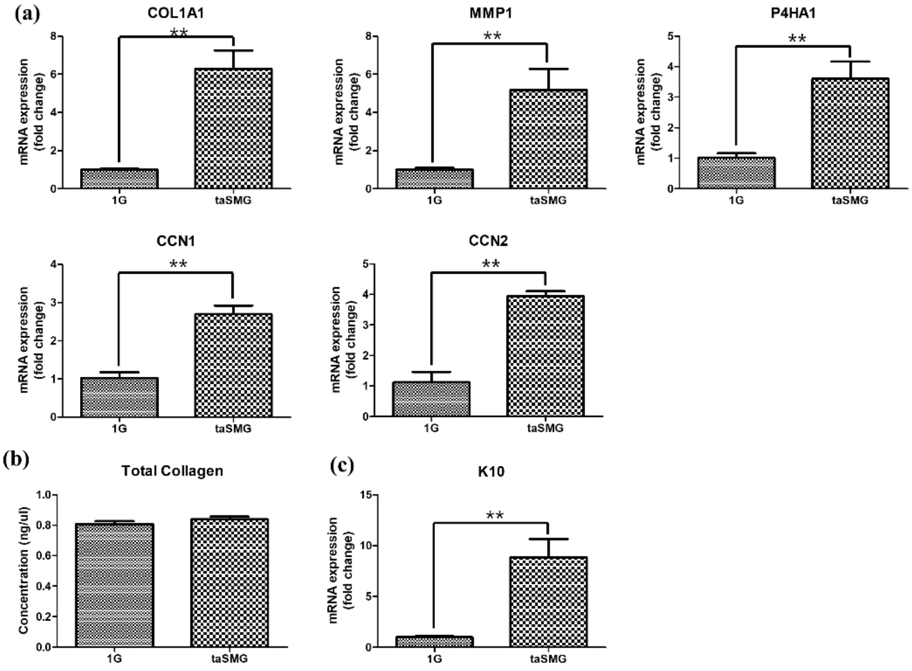

Supplementary Figure 3. Gene expression of co-cultured cells under 1G and taSMG. (a) qRT-PCR analysis of COL1A1, MMP-1, P4HA1, CCN1, and CCN2. (b) Hydroxyproline assay result indicating collagen contents in co-cultured cells at day 3. (c) Cytokeratin-10 (K10) expression of co-cultured cells under 1G and taSMG. Data represent the mean ± SEM ($n = 3$, $**p < 0.01$).
