# Supplementary material for: 3D cell culture using a clinostat reproduces microgravity-induced skin changes
Source: NPJ Microgravity. 2021 Jun 1;7:20. doi: 10.1038/s41526-021-00148-6 (PMC8169764; doi:10.1038/s41526-021-00148-6)
Supplement: Supplementary file 2 — Reporting Summary [file 41526_2021_148_MOESM2_ESM.pdf]

nature research

Corresponding author(s): Sungwan Kim

Last updated by author(s): May 8, 2021

Reporting Summary

Nature Research wishes to improve the reproducibility of the work that we publish. This form provides structure for consistency and transparency in reporting. For further information on Nature Research policies, see our Editorial Policies and the Editorial Policy Checklist.

Statistics

For all statistical analyses, confirm that the following items are present in the figure legend, table legend, main text, or Methods section.

n/a	Confirmed	
☐	☒	The exact sample size (*n*) for each experimental group/condition, given as a discrete number and unit of measurement
☐	☒	A statement on whether measurements were taken from distinct samples or whether the same sample was measured repeatedly
☐	☒	The statistical test(s) used AND whether they are one- or two-sided<br>*Only common tests should be described solely by name; describe more complex techniques in the Methods section.*
☐	☒	A description of all covariates tested
☒	☐	A description of any assumptions or corrections, such as tests of normality and adjustment for multiple comparisons
☐	☒	A full description of the statistical parameters including central tendency (e.g. means) or other basic estimates (e.g. regression coefficient) AND variation (e.g. standard deviation) or associated estimates of uncertainty (e.g. confidence intervals)
☐	☒	For null hypothesis testing, the test statistic (e.g. *F*, *t*, *r*) with confidence intervals, effect sizes, degrees of freedom and *P* value noted<br>*Give P values as exact values whenever suitable.*
☒	☐	For Bayesian analysis, information on the choice of priors and Markov chain Monte Carlo settings
☒	☐	For hierarchical and complex designs, identification of the appropriate level for tests and full reporting of outcomes
☒	☐	Estimates of effect sizes (e.g. Cohen's *d*, Pearson's *r*), indicating how they were calculated

Our web collection on statistics for biologists contains articles on many of the points above.

Software and code

Policy information about availability of computer code

Data collection	NIS Elements, version: 4.60<br>LasX 3.5.7<br>SoftMax Pro Software 5.4.4.007<br>SoftMax Pro 7.1<br>Bio-Rad CFX Maestro 1.1, version: 4.1.2422.1219
Data analysis	All numeric data were processed using Microsoft Excel 2013 (Redmond, WA, USA) and analyzed using SPSS Statistics version 25 software (SPSS, Inc., Chicago, IL, USA). Images were quantitatively analyzed using ImageJ 1.53c software. Values were plotted using GraphPad Prism 5.

For manuscripts utilizing custom algorithms or software that are central to the research but not yet described in published literature, software must be made available to editors and reviewers. We strongly encourage code deposition in a community repository (e.g. GitHub). See the Nature Research guidelines for submitting code & software for further information.

Data

Policy information about availability of data

All manuscripts must include a data availability statement. This statement should provide the following information, where applicable:

- Accession codes, unique identifiers, or web links for publicly available datasets
- A list of figures that have associated raw data
- A description of any restrictions on data availability

All data generated or analyzed during this study are included in this published article or if not are available from the corresponding author on reasonable request.

Field-specific reporting

Please select the one below that is the best fit for your research. If you are not sure, read the appropriate sections before making your selection.

☒ Life sciences ☐ Behavioural & social sciences ☐ Ecological, evolutionary & environmental sciences

For a reference copy of the document with all sections, see nature.com/documents/nr-reporting-summary-flat.pdf

Life sciences study design

All studies must disclose on these points even when the disclosure is negative.

Sample size	No sample-size calculation was performed.
Data exclusions	No data were excluded from the analyses.
Replication	All experiments were repeated at least 3 times.
Randomization	Samples were assigned randomly to 1G and taSMG groups.
Blinding	N/A. This is a comparative study between the 1G group and the taSMG group to investigate the effect of taSMG on cells.

Reporting for specific materials, systems and methods

We require information from authors about some types of materials, experimental systems and methods used in many studies. Here, indicate whether each material, system or method listed is relevant to your study. If you are not sure if a list item applies to your research, read the appropriate section before selecting a response.

Materials & experimental systems

n/a	Involved in the study	
☐	☒	Antibodies
☐	☒	Eukaryotic cell lines
☒	☐	Palaeontology and archaeology
☒	☐	Animals and other organisms
☒	☐	Human research participants
☒	☐	Clinical data
☒	☐	Dual use research of concern

Methods

n/a	Involved in the study	
☒	☐	ChIP-seq
☒	☐	Flow cytometry
☒	☐	MRI-based neuroimaging

Antibodies

Antibodies used	rabbit anti-collagen I (Abcam, ab34710), mouse anti-cytokeratin 10 (K10) (Thermo Fisher Scientific, MA5–13705), goat anti-mouse Alexa Fluor 647 (Abcam, ab150115), and goat anti-rabbit Alexa Fluor 647 (Abcam, ab150075)
Validation	ab34710, ab150115, ab150075: The Abpromise guarantee<br><br>MA5-13705 targets Cytokeratin 10 in IF, IHC (P), and WB applications and shows reactivity with Canine, Feline, Human, and Rat samples.

Eukaryotic cell lines

Policy information about cell lines

Cell line source(s)	Addexbio and ATCC
Authentication	Authentication was done by the supplier.
Mycoplasma contamination	All cell lines tested negative for mycoplasma contamination.
Commonly misidentified lines (See ICLAC register)	N/A
